# Density functional theory studies on N_4_ and N_8_ species: Focusing on various structures and excellent energetic properties

**DOI:** 10.3389/fchem.2022.993036

**Published:** 2022-09-08

**Authors:** Qing Lang, Qiuhan Lin, Pengcheng Wang, Yuangang Xu, Ming Lu

**Affiliations:** School of Chemical Engineering, Nanjing University of Science and Technology, Nanjing, Jiangsu, China

**Keywords:** ploynitrogen, high-energy-density materials, DFT calculation, energetic performance, propellant

## Abstract

All-nitrogen materials, as a unique branch of energetic materials, have gained huge attentions, of which *cyclo-N*_*5*_^*−*^ derivatives are the representative synthetically reported materials. However, the energetic performance of *cyclo-N*_*5*_^*−*^ compounds has certain limitations and cannot go beyond that of CL-20. In order to reach the higher energy, in this work, we presented two kinds of polynitrogen species, N_4_ and N_8_. Two isomers of N_4_ and four isomers of N_8_ were fully calculated by using density functional theory (DFT). Theoretical results show that all these polynitrogen materials exhibit excellent heats of formation (7.92–16.60 kJ g^−1^), desirable detonation performance (D: 9766–11620 m s^−1^; *p*: 36.8–61.1 GPa), as well as the remarkable specific impulses (330.1–436.2 s), which are much superior to CL-20. Among them, **N**_**4**_ **-2** (tetraazahedrane) (D: 10037 m s^−1^; *p*: 40.1 GPa; I_sp_: 409.7 s) and cube **N**_**8**_ **-4** (D: 11620 m s^−1^; *p*: 61.1 GPa; I_sp_: 436.2 s) have the highest energetic properties, which are expected to become promising high-energy-density-materials. Moreover, electrostatic surface potentials, Frontier molecular orbitals, infrared spectra, natural bond orbital charges, and weak interactions were also investigated to further understand their relationship between structure and performance.

## Introduction

In modern society, energetic materials (EMs) have played a significant role in both military and civilian fields, such as gas generating agents, propellants, and explosives for bombs and mines (Yin and Shreeve, 2015; Bu et al., 2019). With the continuous innovation and development of EMs, high-energy-density materials (HEDMs) have attracted great interests due to the desirable energy reserve (Zhang et al., 2015; Zhang et al., 2016; Wang et al., 2019). 2,4,6,8,10,12-hexanitro-2,4,6,8,10,12-hexaazaisowurtzitane (CL-20) and octanitrocubane (ONC) are two typical HEDMs with excellent energetic performance (CL-20: ρ: 2.04 g cm^−3^, D: 9445 m s^−1^, *p*: 46.7 GPa; ONC: ρ: 1.98 g cm^−3^, D: 10100 m s^−1^, *p*: 50.0 GPa) (Eaton et al., 2000; Lang et al., 2020). Unfortunately, few of the reported CHON high-energy-density materials so far have higher energy than CL-20, let alone ONC.

**SCHEME 1:**
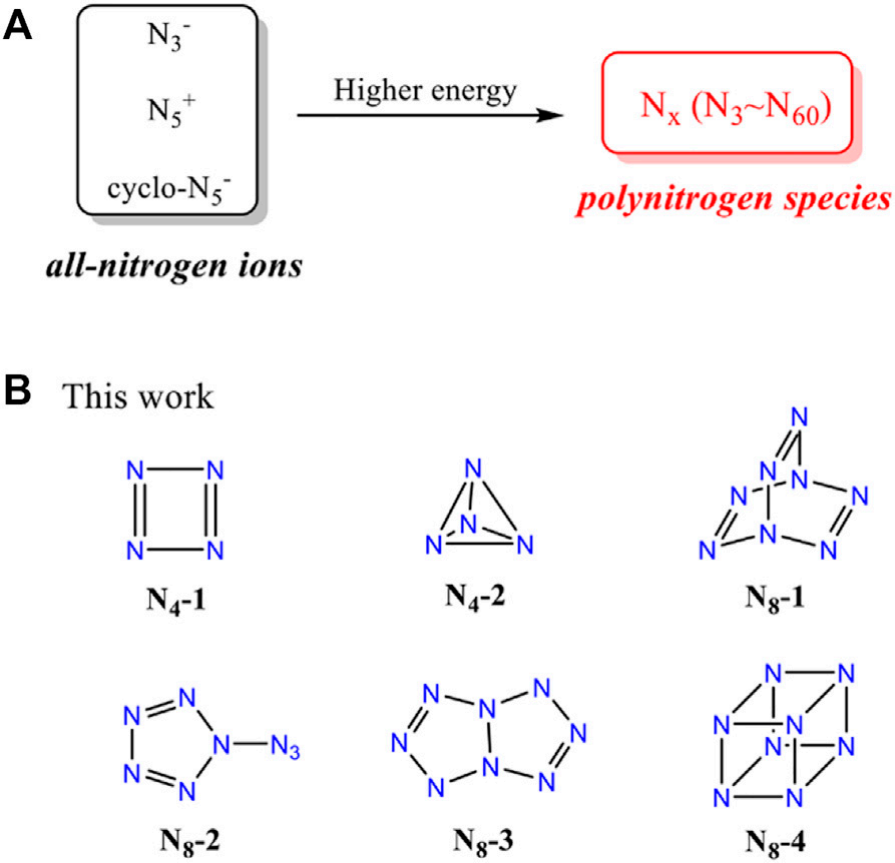
**(A)** The transformation from all-nitrogen ions to pure all-nitrogen materials. **(B)** The structures of N_4_ and N_8_ molecules in this work.

In terms of the above issues, all-nitrogen energetic materials have become a research hotspot, which are able to release huge energy by breaking the N-N (159.9 kJ mol^−1^), N = N (418.2 kJ mol^−1^) bonds in structure and forming N≡N (946 kJ mol^−1^) (Bondarchuk, 2020; Lang et al., 2021). However, the synthesis of all-nitrogen compounds is very difficult, resulting in extremely slow development. To date, only azido anion (N_3_^−^), pentanitrogen cation (N_5_^+^) and pentazolate anion (cyclo-N_5_^*-*^) are successfully synthesized (Christe et al., 1999; Christe, 2017; Wang et al., 2018). N_5_^+^ was first reported in 1995, and was proven to have a polyline structure. All N_5_^+^-based compounds were found to be very sensitive towards external stimuli and their synthesis needs harsh conditions like anhydrous, oxygen-free environment and ultra-low temperatures (Vij et al., 2001; Wilson et al., 2003). Hence, these materials are difficult to get further applications. On the contrary, cyclo-N_5_^-^ can exist stably at normal pressure and temperature, and the first cyclo-N_5_^-^ salt (N_5_)_6_(H_3_O)_3_(NH_4_)_4_Cl, was reported in 2017 (Zhang et al., 2017). Subsequently, a large number of cyclo-N_5_^-^ energetic materials have been prepared and their energetic properties have also been fully estimated (Yang et al., 2018; Xu et al., 2019; Luo et al., 2020; Xu et al., 2022). It is disappointing that cyclo-N_5_^-^ derivatives do not exhibit the expected high density and high detonation performance, whose energy is merely comparable (or even poorer) to RDX, and could not surpass CL-20 (Lin et al., 2020; Wozniak and Piercey, 2020). Main reason for this lies in the fact that all current pentazolate derivatives have the non-energetic or low-energetic cations (i.e., NH_4_^+^, N_2_H_5_^+^, etc.) in the structures, which affect the whole performance (Xu et al., 2020a). Therefore, to bring energy to a higher level, the transformation from all-nitrogen ions to pure all-nitrogen materials is essential, but also a great challenge (Scheme 1A).

As computational chemistry matures, polynitrogen species (N_x_, x from 3 to 60) are particularly attractive due to the extremely high heats of formation arising from abundant N-N and N = N high-energy bonds (Greschner et al., 2016; Türker, 2019). Meanwhile, the decomposition products of these materials are mostly N_2_, making them become green HEDMs (Wu et al., 2014). For the developments of N_x_, density functional theory (DFT) calculations are helpful methods and widely used by many researches (Chen et al 1999; Arhangelskis et al., 2018). According to the theoretical evaluations, the heats of formation of polynitrogen species reach up to 2–5 kcal g^−1^, and detonation velocities even breakthrough the level of 10000 m s^−1^, which are much superior to CL-20 and ONC (Zarko, 2010; Türker, 2018). Moreover, calculations show that these materials have excellent specific impulses (300–500 s), allowing solid propellants equipped with high energetic efficiency to compete with liquid propellants (Türker, 2019). Thus, they exhibit great prospects and are expected to be next-generation high-energy-density materials.

In this work, we devoted to presenting a systematic study on the structures and properties of N_4_ and N_8_ species. Apart from the chain N_4_ and N_8_ (Samartzis and Wodtke, 2010; Hirshberg et al., 2014), two cyclo-N_4_ isomers and four cyclo-N_8_ isomers are calculated by using DFT methods, including optimized structures, molecular electrostatic potentials, Frontier molecular orbitals, non-covalent interaction, natural bond orbital, as well as energetic properties (Scheme 1B). Our study on the structure-property relationships of N_4_ and N_8_ conformation aims to a better understanding of polynitrogen materials.

## Calculation methods

The molecular optimization and frequency calculations were performed by Gaussian 09 (Frisch et al., 2009) package under DFT-B3LYP method with 6–31++G(d,p) basis set. All of the optimized structures were characterized to be true local energy minimum on potential energy surfaces without imaginary frequencies. The geometrical configurations were optimized with no constraints imposed under default convergence criteria. Based on the optimized structures, the highest occupied molecular orbital (HOMO), the lowest unoccupied molecular orbital (LUMO), and infrared (IR) spectrum were calculated. And by using *Multiwfn* program (Lu and Chen, 2012), the electrostatic surface potential (ESP) and interaction region indicator (IRI) analysis were obtained at the same level of theory based on the optimized structures.

For covalent compounds, the intermolecular interactions within the crystals were considered to improve the accuracy of the density of the molecule (cm^3^ per molecule). So, an equation suggested by Politzer et al., (2009) was used.

$$\rho =\alpha \left[M/V\right]+\beta \left(v\sigma_{\mathrm{to}t^{2}}\right)+\gamma$$ (1)

Here *M* is the molecular mass, and *V*_*m*_ is the volume of the isolated gas phase molecule, *ν* is the balance of charges between positive potential and negative potential on molecular surface, and *σ*_*tot*_^*2*^ is strengths and variabilities of the overall surface potentials. The coefficients α, β, γ are 0.9183, 0.0028, 0.0443, respectively.

The calculation of the detonation performance including detonation velocity (D) and detonation pressure (*p*) was performed with K-J equation proposed by Kamlet and Jacobs (1968).

$$D=1\mathrm{.01}{\left(N{\bar{M}}^{1/2}Q^{1/2}\right)}^{1/2}\left(1+1\mathrm{.3\rho}\right)$$ (2)

$$P=1\mathrm{.558}\rho^{2}N{\bar{M}}^{1/2}Q^{1/2}$$ (3)

where *Q* is heat of detonation, *N* is the number of moles of the gas generated per gram, *M̅* is the average molecular weight of gaseous product, and ρ is the calculated density.In the cases of this study, all the molecules have the characteristic of c ≥ 2a + b/2, so the calculation method of Q, N, and M̅ are:

N=(b+2c+2d)/4M (4)

$$\bar{M}=\mathrm{4M}/\left(b+\mathrm{2c}+\mathrm{2d}\right)$$ (5)

$$\mathrm{Q\times}10^{3}=\left(\mathrm{28}\mathrm{.9b}+\mathrm{94}\mathrm{.05a}+0\mathrm{.239\Delta}H_{f}\right)/M$$ (6)

Where *a*, *b*, *c* and *d* stand for the number of C, H, O, N atoms in the explosive molecule, respectively; *ΔH*_*f*_ is the standard heat of formation of the explosive. In addition, the detonation performances of these compounds were also evaluated by the EXPLO5 V6.05.04 program (Sućeska, 2020).The value of I_sp_ was performed using the NASA Chemical Equilibrium with Applications (CEA) thermochemical code at 6.86 MPa with an expansion ratio 70:1, and the supersonic section ratio is considered to be Ae/At = 10, whereas the initial temperature is 298.15 K (Gordon and McBride, 1994; McBride, 1996).

## Results and discussion

### Molecular structure

**FIGURE 1:**
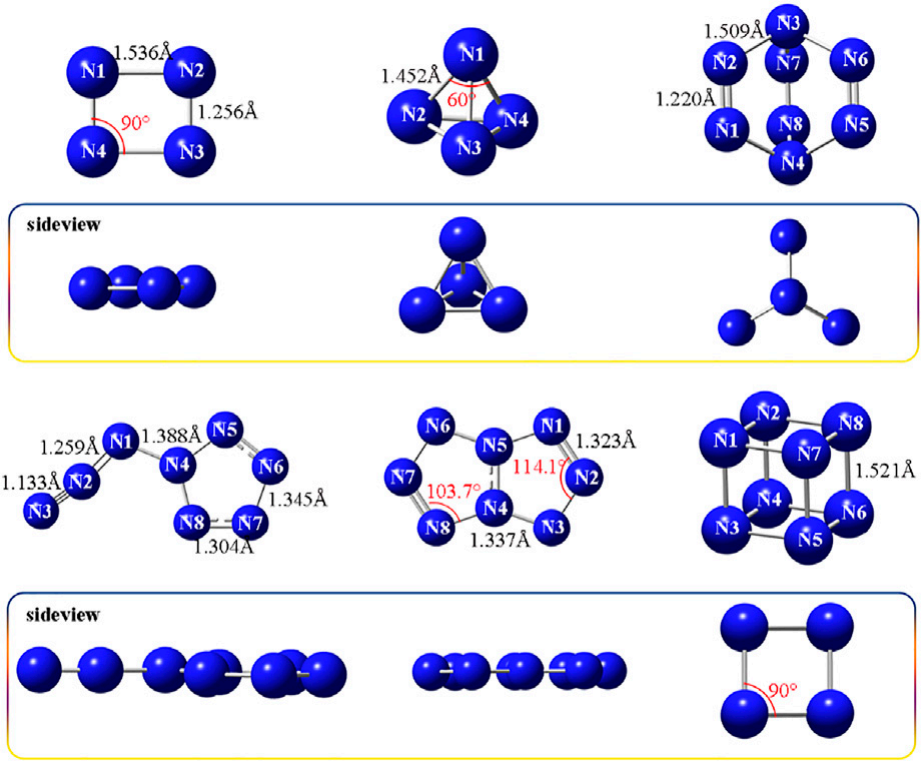
Molecular structures of **N**_**4**_ **-1, N**_**4**_ **-2**, **N**_**8**_ **-1, N**_**8**_ **-2, N**_**8**_ **-3, and N**_**8**_ **-4**, respectively.

The optimized structures of two N_4_ isomers and four N_8_ isomers are presented in Figure 1. The bond lengths of rectangle-shaped **N**_**4**_ **-1** are 1.256 Å (N2-N3) and 1.536 Å (N1-N2), and the bond angles are uniform 90°. **N**_**4**_ **-1** is a flat molecule, which can be evident from the dihedral angles of N4-N1-N2-N3, 0.021 and N1-N2-N3-N4, -0.017. Compound **N**_**4**_ **-2** exhibits a regular tetrahedron structure, with the bond length of 1.452 Å and bond angle of 60, and therefore is known as tetraazahedrane (T_d_N_4_). As for four N_8_ allotropes, **N**_**8**_ **-1**shows a shape similar to a windmill, of which the bond lengths of double bonds (N1 = N2) are 1.220 Å and those of single bonds (N2-N3) are 1.509 Å. While **N**_**8**_ **-2** and **N**_**8**_ **-3** exhibit good planarity, with the dihedral angles of -179.9 (**N**_**8**_ **-2**, N4-N1-N2-N3), -0.02 (**N**_**8**_ **-3**, N5-N4-N3-N2) and 0.06 (**N**_**8**_ **-3**, N5-N4-N8-N7). The N-N bonds distance in **N**_**8**_ **-2** range from 1.133 to 1.388 Å, where N2-N3 of azido group has the shortest value of 1.133 Å. The bond lengths of pentazole ring are 1.345 Å (N7-N8) and 1.304 Å (N6-N7), which are close to those of **N**_**8**_ **-3** (N1-N2, 1.323 Å; N3-N4, 1.337 Å). Similarly, the bond angles of pentazole ring in **N**_**8**_ **-2** and **N**_**8**_ **-3** are both in the range of 103.7°–114.1°. **N**_**8**_ **-4** shows a special cube structure, possessing the bond length of 1.521 Å and bond angle of 90.

In general, the longer bond length may mean the lower stability. For N_4_ isomers, tetraazahedrane (**N**_**4**_ **-2**) has the shorter N-N bonds than rectangle **N**_**4**_ **-1**, suggesting that the three-dimensional tetrahedral structure is conducive to enhancing the molecular stability. In addition to chain N_4_, tetrahedral form of N_4_ has already received growing concerns (Cacace et al., 2002; Nguyen, 2003; Samartzis and Wodtke, 2010). Among the N_8_ isomers, cube N_8_ (**N**_**8**_ **-4**) has the longest bond distance of 1.521 Å. The structure of this material is the analogue of cubane (CH)_8_, which is the skeleton of high-energy-density material ONC. The internuclear N–N–N angles of 90° are far from the standard angle, which is considered to store powerful energy. ONC with remarkable energetic performance is a representative example. However, the longer bond lengths and the unusual bond angles of **N**_**8**_ **-4** may generate the possibility of instability. During subsequent calculations, we found that **N**_**8**_ **-4** has the highest total energy (-437.44 au) among four N_8_ isomers (Supplementary Table S1), and the result is in agreement with the structural features.

### Electrostatic surface potential analysis

**FIGURE 2:**
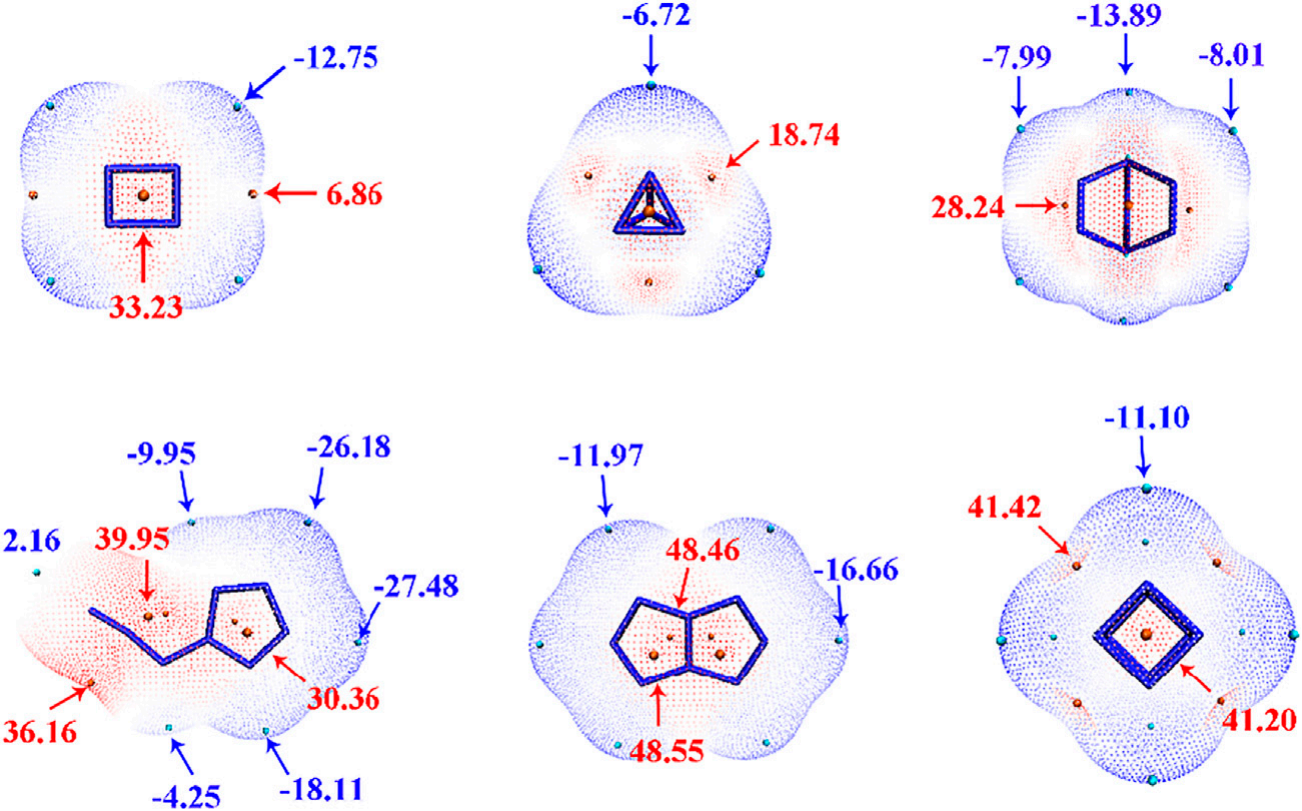
Electrostatic surface potentials mapped molecular vdW surface of **N**_**4**_ **-1, N**_**4**_ **-2, N**_**8**_ **-1, N**_**8**_ **-2, N**_**8**_ **-3,** and **N**_**8**_ **-4**. Significant surface local minima and maxima of ESP are represented as orange and cyan spheres, and labelled by dark blue and red texts, respectively. The unit is in kcal/mol.

**FIGURE 3:**
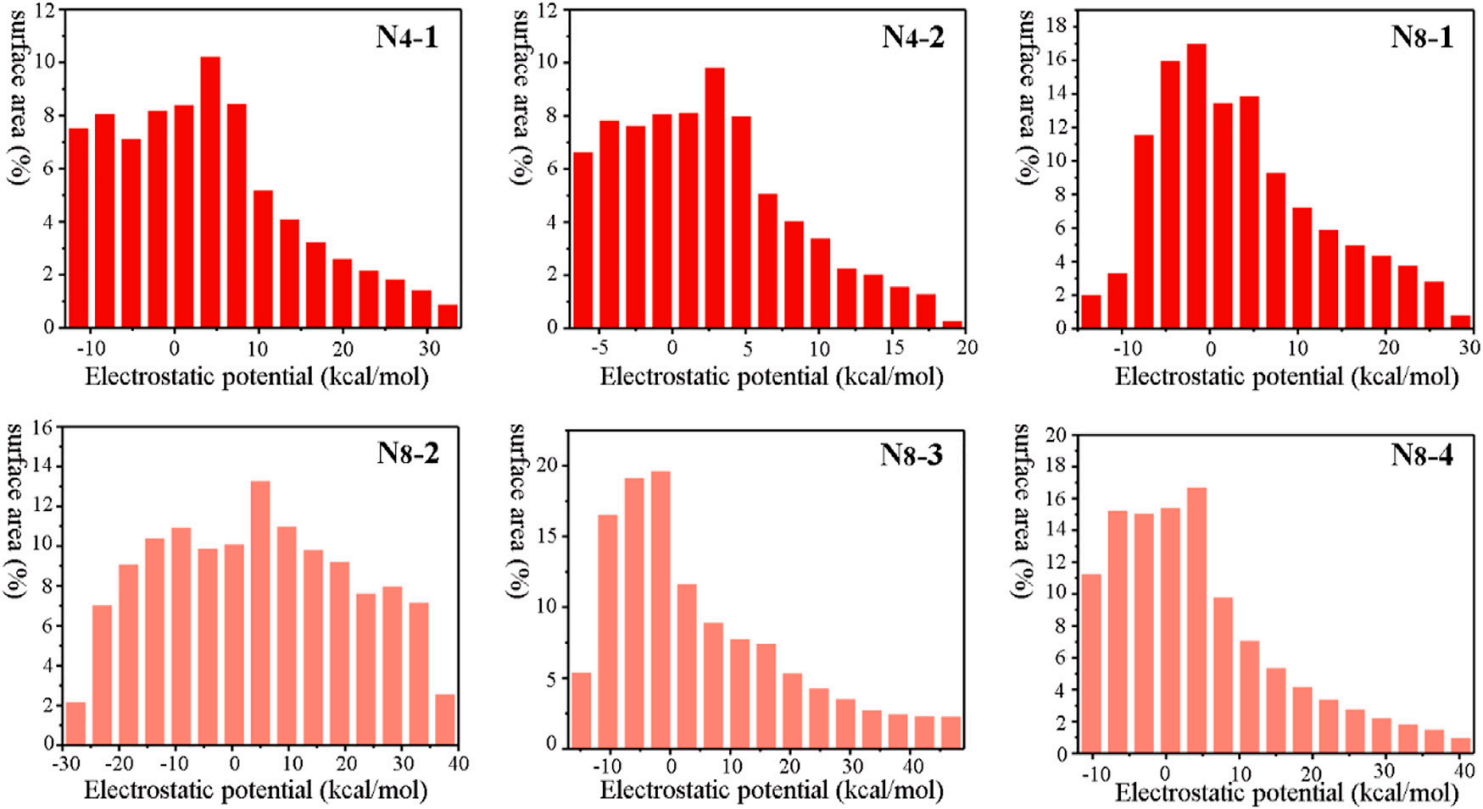
Molecular surface area of **N**_**4**_ **-1, N**_**4**_ **-2, N**_**8**_ **-1, N**_**8**_ **-2, N**_**8**_ **-3, and N**_**8**_ **-4**, respectively.

Electrostatic surface potentials (ESP) of six N_4_ and N_8_ materials were analyzed by Multiwfn program (Manzetti and Lu, 2013). As shown in Figure 2, The minima and maxima of ESP are labeled by blue and red, respectively. The negative electronic potentials in blue indicating the strongest attraction are scattered on the edge of the molecules, while the positive potentials in red representing the strongest repulsion are located at the center of the molecules. Overall, the values of electronic potentials of N_4_ isomers are smaller than those of N_8_ isomers, and **N**_**4**_ **-2** has the lower maximum of ESP with the value of 18.74 kcal mol^−1^ than **N**_**4**_ **-1** (33.23 kcal mol^−1^). As for N_8_ compounds, **N**_**8**_ **-3** and **N**_**8**_ **-4** exhibit the largest maximum surface of ESP with the values of 48.46 kcal mol^−1^ and 41.42 kcal mol^−1^, respectively. The surface minima and maximum of ESP also illustrate the main electrophilic and nucleophile reaction sites, respectively. Meanwhile, molecular surface area in different electrostatic potential intervals was obtained to help us to better understand quantitative distribution of ESP (Figure 3). Among six materials, the distribution of ESP for **N**_**8**_ **-2** is most uniform, while the distributions of ESP for others show the characteristics of “high middle and low sides”.

### Vibration analysis

**FIGURE 4:**
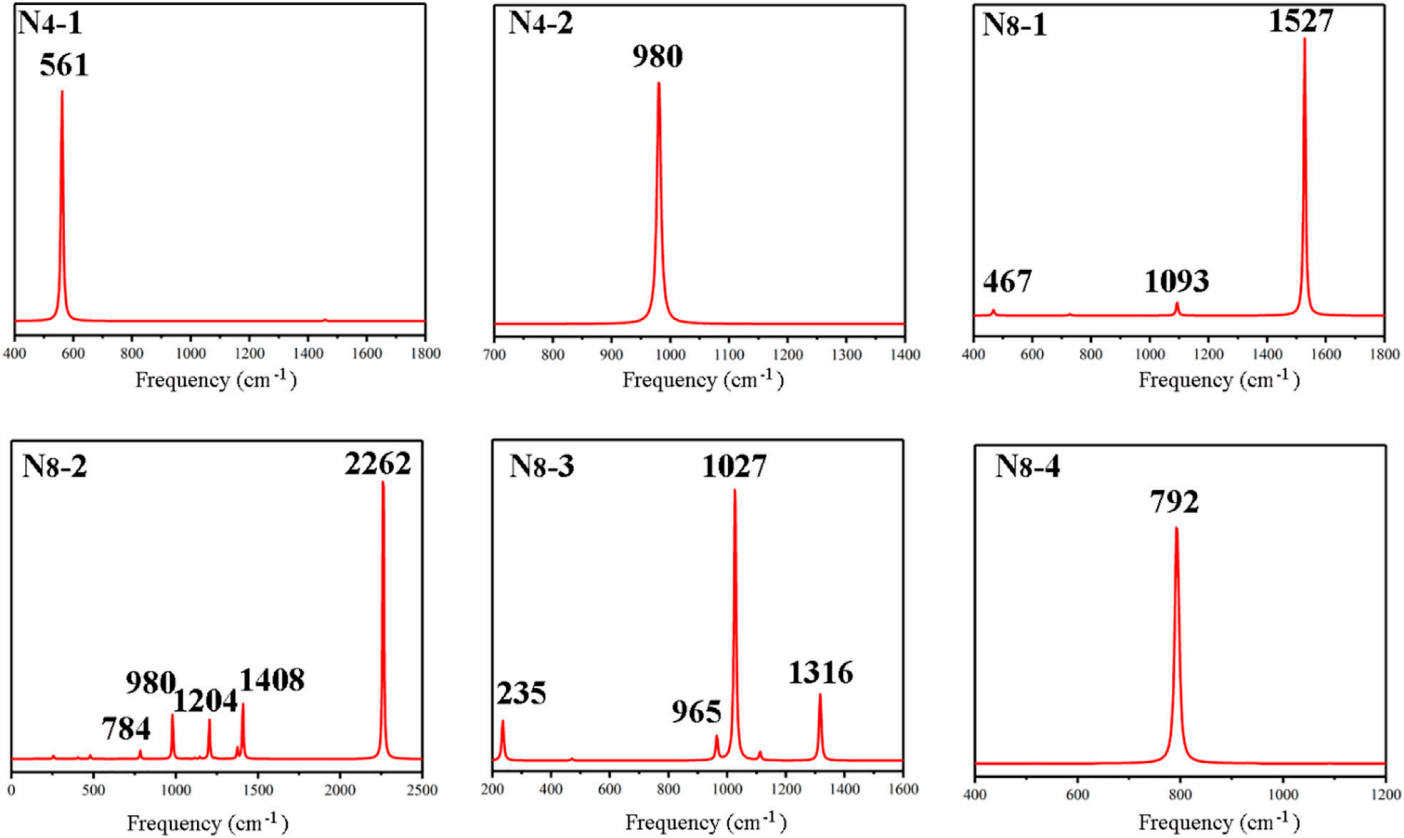
Calculated IR spectra for **N**_**4**_ **-1, N**_**4**_ **-2**, **N**_**8**_ **-1, N**_**8**_ **-2, N**_**8**_ **-3, and N**_**8**_ **-4**, respectively.

The IR spectra of the all-nitrogen compounds were calculated and the results are presented in Figure 4. Both N_4_ isomers show only one typical peak, as well as the cube N_8_, of which the peaks are at 561, 980, 792 cm^−1^, respectively. For other N_8_ isomers, the spectra are more complex. The strong peak at 1527 cm^−1^ of **N**_**8**_ **-1** is assigned to stretching vibration modes of the three N=N bonds in structure, while other two weak peaks at 467 and 1093 cm^−1^ arise from the stretching vibrations of single bonds. As for **N**_**8**_ **-2**, the peaks at 980, 1204, 1408 cm^−1^ result from the stretching vibration modes of pentazole ring, and the strongest absorption at 2262 cm^−1^ arises from the stretching vibrations of N1–N2 and N2–N3 bonds in azide group. Another weak absorption at 784 cm^−1^ is assigned to stretching vibration of N1-N4 bond connecting pentazole and -N_3_. The peak at 235 cm^−1^ in the spectrum of **N**_**8**_ **-3** arises from the torsion vibration of fused N4-N5 bond. And others at 965, 1027, 1316 cm^−1^ belong to the stretching vibrations of two five-membered rings, of which the strongest absorption at 1027 cm^−1^ is stretching vibrations of N1-N2, N7-N8.

### Weak interactions and orbitals analysis

**FIGURE 5:**
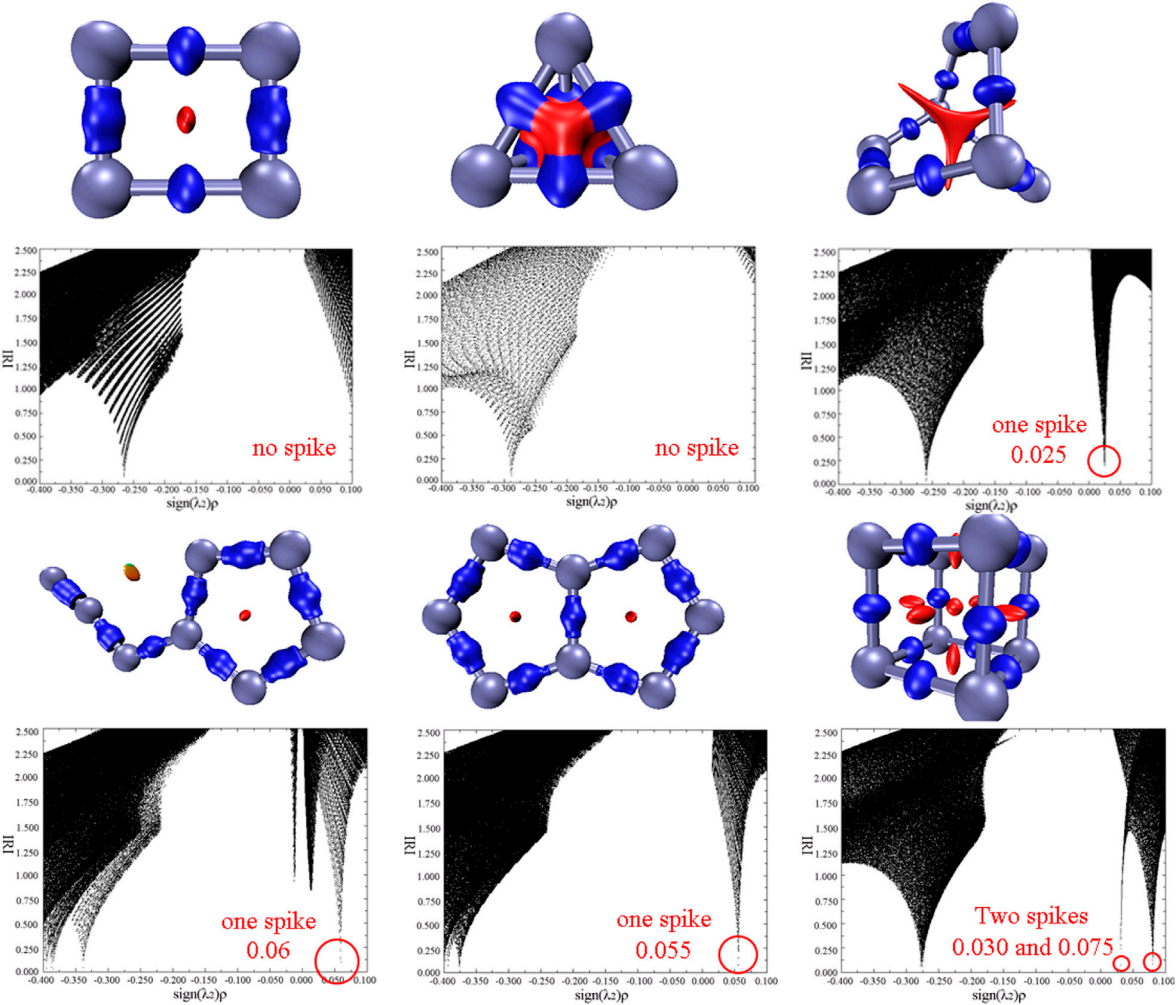
Isosurface maps of IRI= 1.0, and scatter map between IRI and sign(λ_2_)ρ for **N**_**4**_ **-1, N**_**4**_ **-2**, **N**_**8**_ **-1, N**_**8**_ **-2, N**_**8**_ **-3, and N**_**8**_ **-4**, respectively. λ_2_ represents the second largest eigenvalue of Hessian of the electron density, and ρ is the electron density.

Exploring the noncovalent interactions of a molecule is beneficial to help us understand the structural characteristics and stabilities of energetic materials. In this work, interaction region indicator (IRI) analysis as an effective method were adopted and the results are shown in Figure 5 (Lu and Chen, 2021). There are two kinds of interactions in each molecule, which are the chemical bonding interactions in blue isosurface and the steric effects in red. For all nitrogen allotropes, the repulsing interactions are concentrated in the center of the rings. Additionally, the scatter maps between IRI and sign(λ_2_)ρ of all compounds are also obtained. Several spikes in the range of -0.4–-0.25 au of all compounds correspond to the chemical bonding interactions, while the spikes ranging in 0–0.1 au mean the repulsive forces. It can be seen that there is no spike in the scatter plot of two N_4_ isomers, suggesting their weaker repulsions than N_8_ isomers. Among the N_8_ allotropes, the plot of cube N_8_ (**N**_**8**_ **-4**) has two spikes with the value of 0.030 and 0.075 au, and others only respectively have one spike. According to the above analysis, cube N_8_ seems to exhibit the stronger steric effects and thus the poorer stability than other N_8_ conformations.

**FIGURE 6:**
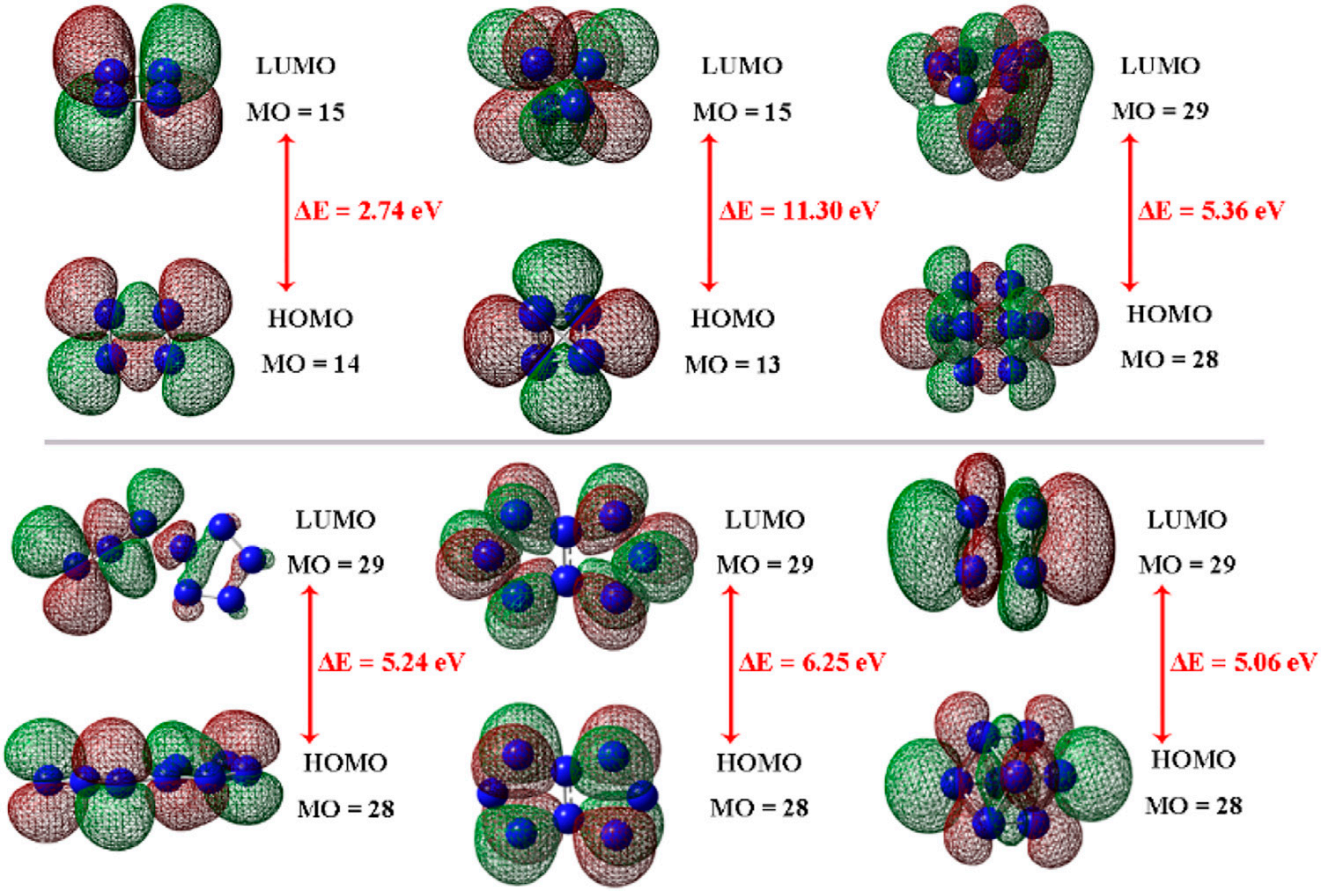
HOMO-LUMO energy levels and energy gaps of **N**_**4**_ **-1, N**_**4**_ **-2, N**_**8**_ **-1, N**_**8**_ **-2, N**_**8**_ **-3,** and **N**_**8**_ **-4,** respectively.

In order to further investigate the molecular stability, the FMOs including highest occupied molecular orbital (HOMO) and lowest unoccupied molecular orbital (LUMO) of all N_4_ and N_8_ isomers were explored. The HOMO and LUMO orbitals and the energy gaps are presented in Figure 6. The red parts represent the positive phase and the green represent the negative phase. The HOMOs and LUMOs are concentrated on each N atom in molecules, except for **N**_**8**_ **-3**, whose HOMOs and LUMOs mainly occupy the unfused N atoms. The HOMOs-LUMOs distributions of these N_4_ and N_8_ materials exhibit good symmetrical features, apart from **N**_**8**_ **-2**. The energy gaps for all materials are 2.74, 11.30, 5.36, 5.24, 6.25, 5.06 eV, respectively. Previous researches have demonstrated that a molecule with better stability may possess higher energy gap (Kosar and Albayrak, 2011; He et al., 2019). Hence, it can be preliminarily judged that tetrahedral-formed N_4_ has superior molecular stability than rectangle one due to the higher energy gaps. For N_8_ isomers, **N**_**8**_ **-3** with fused-ring structure has the largest energy gap and cube-shaped **N**_**8**_ **-4** has the lowest value, revealing the better stability of fused structures and the poor stability of caged materials.

### Natural bond orbital analysis

**FIGURE 7:**
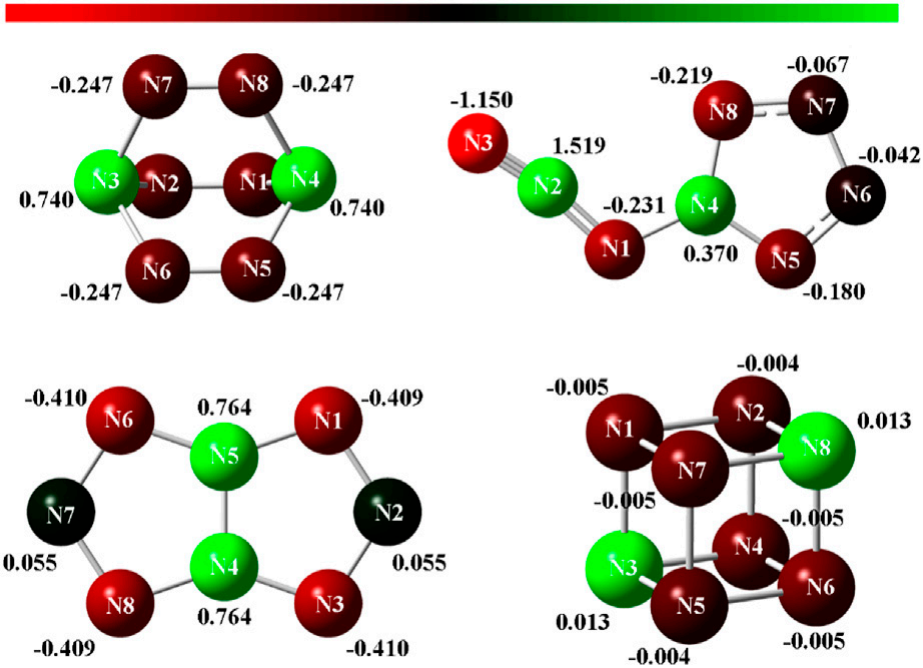
Natural bond orbital charges of **N**_**4**_ **-1, N**_**4**_ **-2, N**_**8**_ **-1, N**_**8**_ **-2, N**_**8**_ **-3, and N**_**8**_ **-4**, respectively.

Natural bond orbital charges (Glendening et al., 2011) of four N_8_ isomers were calculated to understand their charge, bond order, and bond type, and the results are presented in Figure 7. Interestingly, two N atoms in all N_8_ isomers exhibit positive charges, whereas the other six N atoms exhibit negative charges. **N**_**8**_ **-2** shows the most positive charge with the value of 1.519 (N2). For **N**_**8**_ **-1** and **N**_**8**_ **-3**, their distributions of NBO charges are axisymmetric, which are consistent with the characteristics of their structures. And the N atoms in **N**_**8**_ **-3** has the more negative charges than those in **N**_**8**_ **-1**. The negative charges in **N**_**8**_ **-4** are -0.005, which is the lowest among the four N_8_ isomers.

### Energetic properties

In order to accurately predict the detonation properties of all N_4_ and N_8_ isomers, the density and the heat of formation as two important factors should be obtained in advanced. In this study, the densities of all materials were calculated using Equation. 1 proposed by Politzer et, al, and the gas phase heats of formation (Δ_f_H_298K(g)_) were estimated by the atomization approach (Table 2 and Supplementary Table S2). Subsequently, based on the densities and enthalpies, the detonation performance is calculated by both K-J equation and *EXPL O 5* program, which are currently the most widely used computational methods.

As presented in Table 1, because of the more N atoms in structures, N_8_ isomers have much higher densities than N_4_ ones. The density of tetrahedral N_4_ (**N**_**4**_ **-2**) and cube N_8_ (**N**_**8**_ **-4**) are highest, with the values of 1.502 g cm^−3^ and 1.749 g cm^−3^. The densities of other three N_8_ molecules are relatively similar in the range of 1.664–1.697 g cm^−3^. Unfortunately, the densities of these all-nitrogen materials seem no advantages compared to traditional CHON energetic materials, like RDX (1.80 g cm^−3^), HMX (1.91 g cm^−3^) and CL-20 (2.04 g cm^−3^).

**TABLE 1:** B3LYP-6-311++G(d,p) computed properties and calculated densities for N_4_ and N_8_ allotropes.

	M_w_ ^a^ g mol^−1^	V_m_ ^b^ cm^3^ mol^−1^	νσ_tot_^2^ ^c^ kcal mol^−1^	ρ^d^ g cm^−3^
**N**_**4**_ **-1**	56.03	62.579	11.2419	1.441
**N**_**4**_ **-2**	56.03	58.961	3.16614	1.502
**N**_**8**_ **-1**	112.06	107.102	8.71689	1.664
**N**_**8**_ **-2**	112.06	112.357	36.94395	1.669
**N**_**8**_ **-3**	112.06	106.165	15.62189	1.697
**N**_**8**_ **-4**	112.06	101.802	9.21526	1.749

a Molar weight.

b Volume.

c Electro-static potential parameters.

d Density.

The heats of formation (details in Supporting Information) and detonation performance of all compounds are shown in Table 2. It is worth noting that these N_4_ and N_8_ allotropes has very excellent heats of formation of 7.92–16.60 kJ g^−1^, of which cube N_8_ has the highest value of 16.60 kJ g^−1^. This may arise from the numerous N-N and N=N bonds in the structure. The excellent enthalpies of these all-nitrogen materials are much superior to representative CHON explosives, such as RDX (0.36 kJ g^−1^), HMX (0.35 kJ g^−1^) and CL-20 (0.90 kJ g^−1^). In addition, the six poly-nitrogen materials exhibit prominent detonation performance. The values of detonation velocities fall in the range of 9747–11620 m s^−1^, while the detonation pressures are in the range of 36.80–61.10 GPa (calculated by *EXPL O 5*). Among them, N_8_ isomers show higher detonation performance than N_4_ ones due to the higher density. There is no doute that **N**_**8**_ **-4** has the best detonation performance (D: 11620 m s^−1^; *p*: 61.1 GPa). All the all-nitrogen materials presented in this work has much higher energy density than CL-20 (D: 9445 m s^−1^, *p*: 46.7 GPa). And compared to N_5_^+^N_3_^−^ in our previous work (Xu et al., 2020b), **N**_**8**_ **-2** (ρ: 1.669 g cm^−3^, D: 9747 m s^−1^, *p*: 38.0 GPa) exhibits higher density and higher detonation performance based on the same structural composition of pentazole ring and azide (N_5_^+^N_3_^−^: ρ: 1.55 g cm^−3^, D: 9290 m s^−1^, *p*: 34.67 GPa). The above discussion not only confirms that all-nitrogen materials have the great prospects because of the huge energy, but also that covalent poly-nitrogen compounds would be more powerful than all-nitrogen salts.

**TABLE 2:** Physico-chemical properties of N_4_ and N_8_ allotropes compared with RDX, HMX and CL-20.

	Δ_f_H_298K(g)_ ^a^ kJ mol^−1^/kJ g^−1^	D^b^ m s^−1^	P^c^ GPa	I_sp_ ^d^ s
**N**_**4**_ **-1**	768/ 13.71	9543/ 9766	34.98/ 36.80	410.3
**N**_**4**_ **-2**	765.1/ 13.66	9797/ 10037	37.94/ 40.10	409.7
**N**_**8**_ **-1**	1064.6/ 9.50	9586/ 10068	38.84/ 41.50	356.9
**N**_**8**_ **-2**	887.1/ 7.92	9177/ 9747	35.67/ 38.0	330.1
**N**_**8**_ **-3**	945.9/ 8.44	9433/ 10000	38.07/ 40.8	339.4
**N**_**8**_ **-4**	1860.3/ 16.60	11406/ 11620	56.72/ 61.1	436.2
**RDX** **^e^**	70.3/ 0.36	8795	34.9	268.3^f^
**HMX** **^e^**	104.8/ 0.35	9144	39.5	-
**CL-20** **^e^**	397.8/ 0.90	9445	46.7	272.6^g^

a Gas phase heats of formation (g, 298 K).

b Detonation velocity: left, K-J equation; right, EXPLO, 5 V6.05.04.

c Detonation pressure: left, K-J equation; right, EXPLO, 5 V6.05.04.

d Specific impulse.

e Ref. (Lang et al., 2022).

f Ref. 19.

g Ref. 9

In addition to the excellent detonation performance, N_4_ and N_8_ isomers also possess exceptional theoretical specific impulse (I_sp_). The values of I_sp_ for two N_4_ isomers are 410.3 s and 409.7 s, respectively, which are higher than those of **N**_**8**_ **-1** (356.9 s), **N**_**8**_ **-2** (330.1 s), **N**_**8**_ **-3** (339.4 s), but poorer than **N**_**8**_ **-4** (436.2 s). Cube N_8_ in this work combines the best detonation performance and best specific impulse, suggesting the unique advantages of cage structure. And the specific impulses (330.1–436.2 s) of six poly-nitrogen molecules are much superior to that of CL-20 (272.6 s). It is believed that these ploy-nitrogen materials are expected to become the promising candidates as solid rocket propellants.

## Conclusion

In this study, two structures of cyclo-N_4_ (**N**_**4**_ **-1**, **N**_**4**_ **-2**) and four structures of cyclo-N_8_ (**N**_**8**_ **-1**, **N**_**8**_ **-2**, **N**_**8**_ **-3**, **N**_**8**_ **-4**) were designed and calculated through density functional theory methods. The optimized structures, molecular electrostatic potentials, Frontier molecular orbitals, non-covalent interaction, natural bond orbital, and most importantly, the energetic properties of six poly-nitrogen materials were fully investigated. According to the theoretical calculation, **N**_**4**_ **-2** with tetrahedron structure possesses shorter N-N bond lengths than rectangle-shaped **N**_**4**_ **-1**, and thus the higher molecular stability. Cube **N**_**8**_ **-4** has the longest bond lengths and unusual bond angle among N_8_ isomers, which may lead to the poor stability. The results from Frontier molecular orbitals and non-covalent interaction are also consistent with structure analysis. The densities of six compounds are 1.441–1.749 g cm^−3^, and the heats of formation are 7.92–16.60 kJ g^−1^. With such remarkable enthalpies, these N_4_ and N_8_ isomers exhibit excellent detonation performance (D: 9747–11620 m s^−1^; *p*: 36.8–61.6 GPa) and high specific impulses (I_sp_: 330.1–436.2 s), which are superior to CL-20 (D: 9445 m s^−1^; *p*: 46.7 GPa, I_sp_: 272.6 s). In particular, **N**_**4**_ **-2** and **N**_**8**_ **-4** have highest energy density among N_4_ and N_8_ species, respectively, revealing their huge development potential as next-generation HEDMs beyond CL-20.

## Data Availability

The original contributions presented in the study are included in the article/supplementary material, further inquiries can be directed to the corresponding authors.
