# Supplementary material for: Density functional theory studies on N4 and N8 species: Focusing on various structures and excellent energetic properties
Source: Front Chem. 2022 Sep 8;10:993036. doi: 10.3389/fchem.2022.993036 (PMC9492962; doi:10.3389/fchem.2022.993036)
Supplement: Supplementary file 1 [file DataSheet1.docx]

**Density functional theory studies on N_4_ and N_8_ species: Focusing on various structures and excellent energetic properties**

**Qing Lang^1^, Qiuhan Lin^1^, Pengcheng Wang^1^, Yuangang Xu^1*^, Ming Lu^1*^**

^1^School of Chemical Engineering, Nanjing University of Science and Technology, Xiaolingwei 200, Nanjing, Jiangsu, China.

1. **Calculated section of all N_4_ and N_8_ species**

**Table S1** Calculated heat of formation of six compounds.

| Compd | E_0_/au | ZPE/au | H_T_/au | **H_f_/ kJ mol^-1^** | **H_f_/ kJ g^-1^** |
| --- | --- | --- | --- | --- | --- |
| **N_4_-1** | -217.7810333 | 0.013972 | 0.004294 | **768.04** | **13.71** |
| **N_4_-2** | -218.781042 | 0.013009 | 0.004131 | **765.06** | **13.66** |
| **N_8_-1** | -437.7041522 | 0.029159 | 0.006116 | **1064.64** | **9.50** |
| **N_8_-2** | -437.8143231 | 0.035445 | 0.007101 | **887.09** | **7.92** |
| **N_~~8~~_-3** | -437.7918126 | 0.036645 | 0.005809 | **945.95** | **8.44** |
| **N_8_-4** | -437.4400863 | 0.033597 | 0.005382 | **1860.28** | **16.60** |

**Table S2** Calculated detonation performance of six compounds by K-J equation.

| Compd | N | M̅ | Q.10^-3^ | **D/ m s^-1^** | **P/ GPa** |
| --- | --- | --- | --- | --- | --- |
| **N_4_-1** | 0.035695163 | 28.015 | 3.275 | **9543** | **34.98** |
| **N_4_-2** | 0.035695163 | 28.015 | 3.262 | **9797** | **37.94** |
| **N_8_-1** | 0.035695163 | 28.015 | 2.270 | **9586** | **38.84** |
| **N_8_-2** | 0.035695163 | 28.015 | 1.892 | **9177** | **35.67** |
| **N_8_-3** | 0.035695163 | 28.015 | 2.017 | **9433** | **38.07** |
| **N_8_-4** | 0.035695163 | 28.015 | 3.967 | **11406** | **56.72** |

1. **Distributions of electrostatic potentials**

**
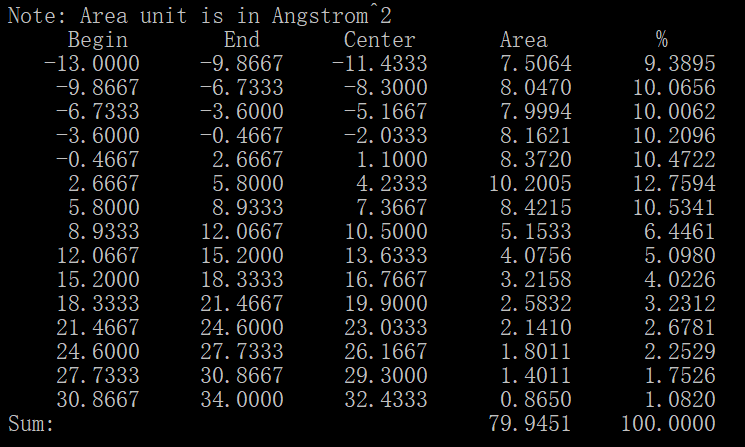
**

**Figure S1.** Distributions of electrostatic potentials of **N_4_-1**.


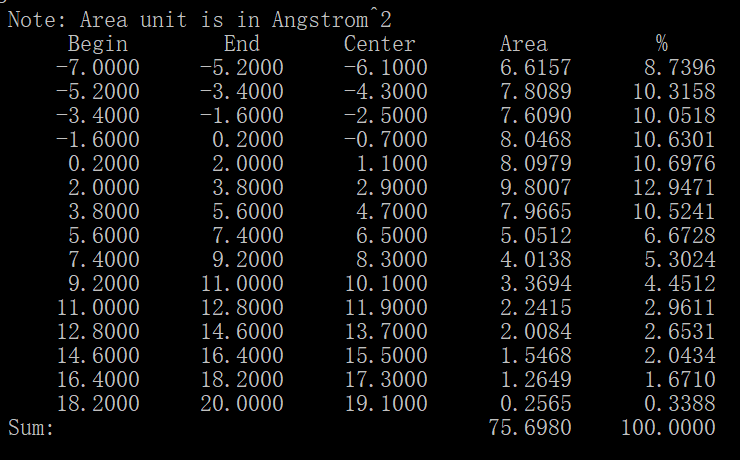


**Figure S2.** Distributions of electrostatic potentials of **N_4_-2**_._


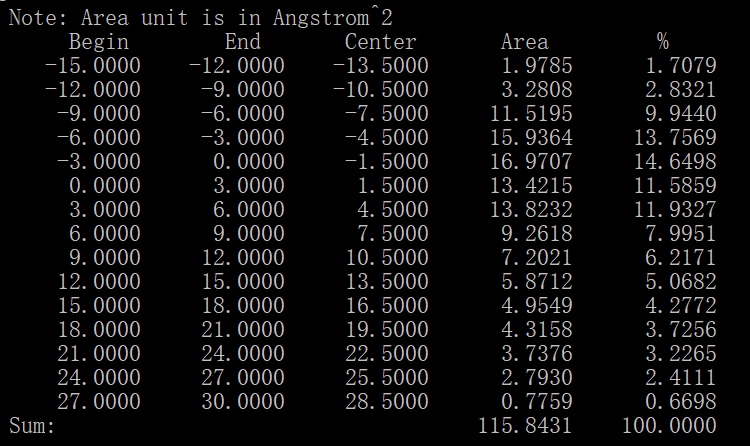


**Figure S3.** Distributions of electrostatic potentials of **N_8_-1**.


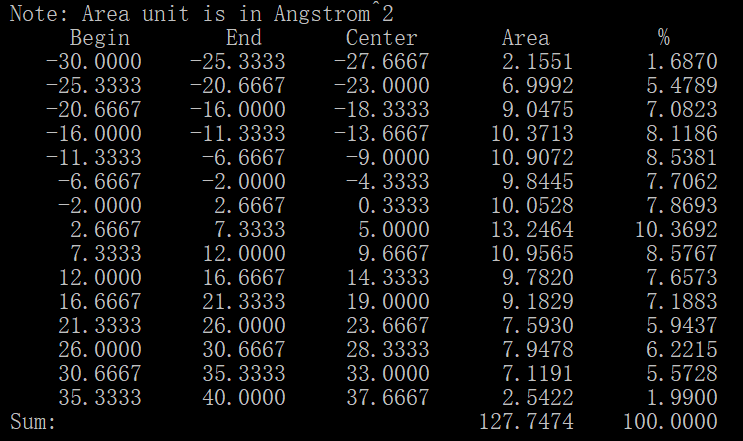


**Figure S4.** Distributions of electrostatic potentials of **N_8_-2.**

**
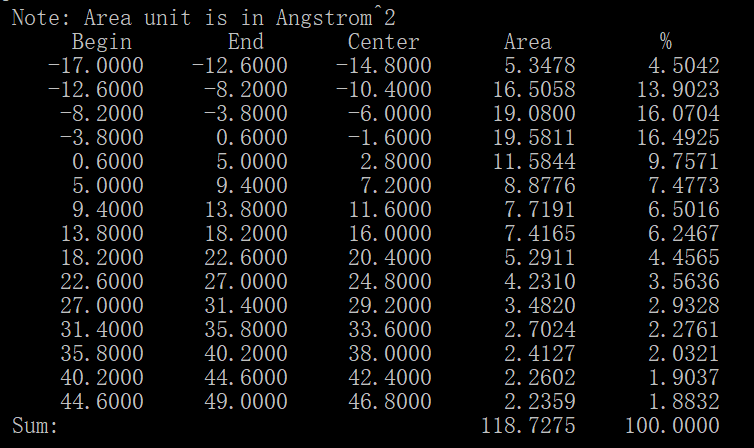
**

**Figure S5.** Distributions of electrostatic potentials of **N_8_-3.**

**
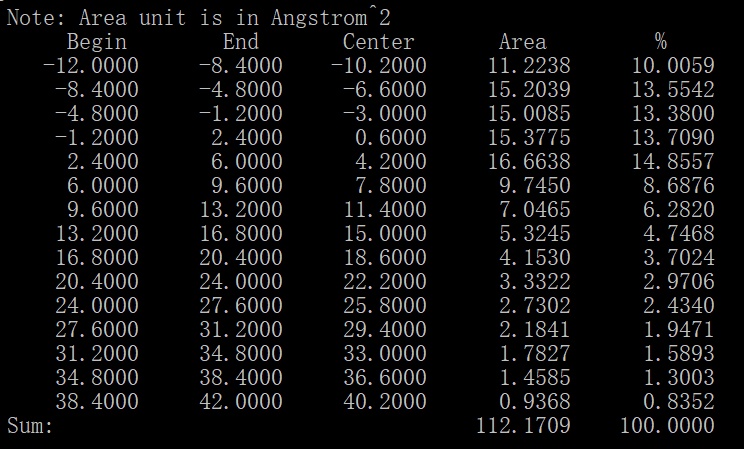
**

**Figure S6.** Distributions of electrostatic potentials of **N_8_-4.**
